# Recombinant Yeast-Based Vaccines: Importance and Applications

**DOI:** 10.3390/idr17050118

**Published:** 2025-09-18

**Authors:** Ravinder Kumar

**Affiliations:** Department of Clinical Pharmacy and Translational Science, University of Tennessee Health Science Center, Memphis, TN 38163, USA; raj86tau@gmail.com

Vaccines are biological preparations used to elicit an immune response, in order to prevent future infections or minimize damage from possible future infection [1]. Vaccine development initially followed a classical approach, utilizing attenuated and/or killed pathogens [1]. In contrast, modern vaccine development employs a diverse range of platforms, tailored to the specific needs and pathogens involved. For example, where possible, classic methods of vaccine manufacturing are still used (possible because pathogens or associated organisms can be grown commercially), involving only a few components of the pathogen (sometimes only one), which may include protein/peptide, nucleic acid, or carbohydrate moiety, overcoming the issue of large-scale culture of pathogen commercially (example protozoa *P. falciparum*, bacteria like *M. leprae*) [2,3,4]. Vaccines save millions of lives every year [5]. It is only due to the widespread use of vaccines that we can control the COVID-19 pandemic and minimize loss of life due to SARS-CoV-2 (severe acute respiratory syndrome coronavirus 2) infection [6].

The COVID-19 (Coronavirus disease 2019) pandemic opened the door for the use and acceptance of mRNA-based vaccines. This also led to the development of other mRNA-based vaccines against different diseases [7]. Like mRNA or DNA-based vaccines (example of nucleic acid-based vaccines), recombinant yeast-based vaccines are making their presence known in both research and clinical settings. For example, surface protein expressed and purified from yeast (*S. cerevisiae*) is regularly used to prevent hepatitis B virus infection [8]. Similarly, virus-like particles or VLPs (of HPV16) also expressed and purified from *S. cerevisiae* have been used to avoid human papillomavirus (HPV) infection [9]. Apart from these, several other protein antigens are expressed and purified from yeast (to be used as vaccines) and are in different phases of clinical trials or waiting for FDA approval. The GRAS (Generally Recognized As Safe) status of yeast, particularly *S. cerevisiae*, and the successful use of peptides/proteins purified from several yeasts to be used as vaccines demonstrates that yeast is a safe and reliable platform for vaccine development [10].

Other salient features that make use of yeast as a potential platform for vaccine development are discussed briefly. The use of whole recombinant yeast (WRY) or yeast surface display (YSD) was able to prompt a safe and specific immune response in preclinical studies [11,12]. These approaches prevent the purification of expressed peptides or proteins, thereby making vaccine production faster and cheaper. The use of these two platforms precludes the use of a separate adjuvant, as the yeast cell wall acts as a natural adjuvant, which again makes vaccine formulation simpler and cheaper [13]. The use of whole recombinant yeast can also help in developing a thermostable vaccine. For example, some studies have shown that protein antigens remain stable (in the cellular environment of yeast) for more than a year at room temperature and at 37 °C [14,15]. Furthermore, it was also shown that within yeast cells (lyophilized yeast powder), expressed immunogens remain stable even after freeze–thaw [15]. The use of whole recombinant yeast (or intact yeast cells) can induce cell-mediated immunity, further making them the platform of choice [16,17].

The application of inactivated yeast in healthy human subjects was found to be safe, opening the door for the use of inactivated whole yeast cells in the human population [18]. However, it should be noted that some people may have an allergic reaction to yeast, its products, or derivatives, and therefore such information must be considered before administering yeast-based vaccines [19]. The observation that the level of immune response mounted is independent of the live or dead nature has significant implications, as inactivated recombinant yeast prevents the use of live yeast as a vaccine without compromising on immune response [20]. This is also important because it allows for the long-term storage of yeast powder at room temperature. Unlike the inactivation of pathogens with chemicals like formalin, which is fast, safe, and easy, yeast can be inactivated by incubating cells at 56 °C for 90 min. One need not worry about residual formalin in the vaccine [14,15].

Apart from the use of internal administration (in the form of injection), the whole recombinant yeast can also be used as an edible vaccine or oral application [11,21]. This oral application or feeding of whole recombinant yeast (vaccine) can have significant implications, especially in veterinary medicine. The edible nature of yeast-based vaccines allows for the mixing of recombinant yeast powder with feed or its addition to water used for drinking by farm animals or birds. This will facilitate mass vaccination on farms without the need for trained personnel, as the powdered form of recombinant yeast cells allows for safe storage without the requirement for special cold chain facilities [15,22,23].

As mentioned above, yeast offers several options for vaccine development. There is still another essential feature that makes yeast a choice platform for vaccine development or production. Unlike plants, recombinant yeast can be grown under controlled environmental conditions, which means a lower risk of escape into the environment, unlike plants grown in polyhouses or in open fields [24]. Managing the waste products of yeast (which are mostly spent or used media) is also easy. Unlike the bacterial system, yeast, due to its eukaryotic nature, can undergo post-translational modification (PTM) of the expressed protein antigen. This is important as the PTM of the expressed protein immunogen can have a significant effect on the level of immune response [25]. Unlike mammalian cells, the media required to culture yeast cells are cheap, and yeast cells can be grown in bulk in an industrial-scale fermenter (especially species like *P. pastoris*) [24].

The real beauty of using yeast for vaccine development lies in the fact that the same system can be used in different ways, including through the expression and purification of protein or peptide antigens, virus-like particles, whole recombinant yeast (WRY), and yeast surface display (YSD). WRY/YSD can be used as edible or oral applications as well as for internal applications [11].

Given the rapid and continuous increase in fungal burden, along with the widespread emergence of antifungal resistance to common antifungal drugs, there is a need for a better approach to managing fungal infections [26]. This is likely achievable with a yeast-based approach. Several studies in the past have shown that the use of inactivated yeast or recombinant yeast expressing the antigen of interest was able to prompt a specific antifungal immune response and provide significant protection [27]. Since the cell wall composition is quite similar across different pathogenic fungal species, people in the community are proposing a pan-antifungal vaccine in which the application of one pathogenic yeast or even a nonpathogenic yeast (like budding yeast) may be able to provide the required protection against broad fungal infection. Apart from encouraging results against infectious diseases, yeast-based vaccines are also being evaluated for their potential to treat cancer [28,29].

Therefore, considering all these factors, the use of a yeast-based platform for vaccine development holds great interest and potential. This platform can also overcome challenges such as the need for a cold chain for vaccine distribution or dissemination to the final user. It is also essential that both labs and industry should develop and follow a standard guideline for developing yeast-based vaccines [30]. The actual value of this simple approach can be appreciated by looking at Table 1, showing different vaccine formulations in which the immunogen was expressed and purified from yeast.

## Figures and Tables

**Table 1:** Yeast-derived vaccines which have been approved for commercial use by the FDA or are presently in clinical use [24].

Trade Name	Infectious Agent	Disease	Company	Antigen	Type	Status
Gardasil^®^	HPV	Cervical carcinoma	Merck (Durham, NC)	L1	VLPs	Approved
Gardasil9^®^	HPV	Cervical carcinoma	Merck (Durham, NC)	L1		Approved
Mosquirix™	*P. falciparum*	Malaria	Univ. of Rochester AVEG, Rochester, NY, USA	*P. falciparum* circumsporozoite protein fused to the Hepatitis B surface antigen	VLPs	Approved
	HIV	Acquired immune deficiency syndrome (AIDS)		HIV p17/p24: Ty-VLP	VLPs	Clinical trial phase 1 (Clinical trial No.: NCT00001053) [31]
Hepavax-Gene	HBV	Hepatocellular carcinoma	Crucell (Dusseldorf, Germany)	SHBs, MHBs	VLPs	Licensed
Fendrix	HBV	Hepatocellular carcinoma	GSK (Rixensart, Belgium)	SHBs	VLPs	Licensed
Heplisav-B	HBV	Hepatocellular carcinoma	Dynavax (Oakland, CA, USA)	SHBs	VLPs	Licensed
Engerix	HBV	Hepatocellular carcinoma	GSK (London, UK)	SHBs	VLPs	Licensed
Recombivax HB (H-B-Vac^®^-II)	HBV	Hepatocellular carcinoma	Merck Vaccine (Kirkland, QC, Canada)	SHBs	VLPs	Licensed

HPV: human papillomavirus, HIV: human immunodeficiency virus, HBV: hepatitis B virus, VLPs: Virus-like particles, GSK: GlaxoSmithKline, UK: United Kingdom, SHBs: small hepatitis B virus surface antigen, MHBs: hepatitis B virus middle surface antigen.
